# Effect of iodine supplementation in pregnancy on neurocognitive development on offspring in iodine deficiency areas: a systematic review

**DOI:** 10.20945/2359-3997000000376

**Published:** 2021-06-29

**Authors:** Almeida A. L. Machamba, Francilene M. Azevedo, Karen O. Fracalossi, Sylvia do C. C. Franceschini

**Affiliations:** 1 Universidade Federal de Viçosa Departamento de Nutrição e Saúde Viçosa MG Brasil Departamento de Nutrição e Saúde, Universidade Federal de Viçosa (UFV), Viçosa, MG, Brasil

**Keywords:** Potassium iodide, cognition, child, pregnant woman

## Abstract

**Objective:**

To investigate the effect of iodine supplementation during gestation on the neurocognitive development of children in areas where iodine deficiency is common.

**Materials and methods:**

Based on the PRISMA methodology, we conducted the search for articles in the PubMed, LILACS and Scopus databases, between March and April 2020, without limitation of dates. We used descriptors in English, Portuguese, and Spanish, without filters. Four clinical trials and four cohort articles were included in the review.

**Results:**

The maximum supplementation was 300 μg of potassium iodide per day. The Bayley scale and Children’s Communication Checklist-Short were used to assess neurodevelopment in children. There was no significant improvement in the children’s mental development index and behavioural development index in the supplemented group; however, the psychomotor development index (PDI) showed improvement in the poorer gross motor skills. We found differences in the response time to sound in the supplemented group living in mild deficiency areas.

**Conclusion:**

Daily supplementation with iodine can improve poor psychomotor development of children living in mild to moderate iodine deficiency areas. Thus, it is necessary to perform further studies to assess the effect of supplementation on neurodevelopment before, during and after gestation in mild to moderate iodine deficiency areas.

## INTRODUCTION

Iodine deficiency affects almost 2 billion people worldwide (1). In 2017, 18 countries were identified in which women of reproductive age were iodine-deficient, whereas for pregnant women, this was found in 39 countries (2). At this stage, deficiency induces the occurrence of irreversible brain damage in children (1). In fact, inadequate iodine intake in the foetal period may cause dwarfism, cretinism, mental retardation, deafness, psychomotor defects, or congenital anomalies, and may lead to miscarriage or stillbirth (3). Throughout growth, it negatively affects physical and neurocognitive development, especially hippocampal development and memory functions, and in adult life, causes goiter and hypothyroidism (4).

The recommended daily intake of iodine is 90 μg in the age group 0-59 months, 120 μg in 6-12-year-olds, 150 μg in adolescents and adults, and 250 μg during gestation and lactation (5). To ensure sufficient iodine intake, women who are planning pregnancy, pregnant or lactating should be recommended by the American Thyroid Association and European Thyroid Association to ingest daily oral supplements containing 150 μg of iodine (6,7). The World Health Organization (WHO) affirm that this supplementation should be undertaken when iodized salt does not reach over 90% of households (5).

Recent findings in mild iodine deficiency areas in Israel and Iceland report the improvement of iodine intake in pregnant women supplemented with iodine compared with those not taking iodine supplements (8,9). Other studies in mild iodine deficiency areas in Brazil showed that supplementation corrects maternal thyroid indices and avoids impairment of the neuropsychological development in the offspring (10).

However, the effectiveness of iodine supplementation in pregnant women at improving children’s cognitive development is poorly explored and uncertain (11-13). Therefore, this review aimed to investigate the effect of iodine supplementation during gestation on children’s neurocognitive development in iodine deficiency areas.

## MATERIALS AND METHODS

This systematic review sought to answer the following question: “What is the effect of iodine supplementation during gestation on children’s cognitive development?”. The review protocol was registered in PROSPERO (International Prospective Register of Ongoing Systematic Reviews) with the identification number CRD42019116962.

We used the PRISMA (Preferred Reporting Items for Systematic Reviews and Meta-Analyses) (14) methodology to select articles. To identify the articles, we conducted the search in the PubMed, LILACS (Health Sciences in Latin America and the Caribbean) and Scopus databases, from March 1st to April 1st 2020, without limitations of dates. We used the descriptors: “iodine AND supplementation AND child AND development AND cognitive”, provided by DeCS (Health Science Descriptors) (15), in English, Portuguese, and Spanish, without filters (Supplement Appendix 1).

After the searches and elimination of duplicates by database and between databases, we registered all articles in a spreadsheet in Microsoft Excel^®^. Then, we recorded data from the articles, detailing the year, authorship, place of origin, type of study, target population, sample size, dose and time of supplementation, tests to assess neurocognitive development, and main results observed.

The inclusion criteria were that the studies should be randomized or non-randomized controlled trials or cohorts that evaluated the effect of iodine supplementation during gestation on the neurocognitive development of children living in moderate to severe, mild to moderate, severe, moderate, or mild iodine deficiency regions. We included all children in this study, without any age limit, provided that the study presented some scale of measurement of their neurodevelopment. Studies on the effect of intake of fortified foods, as well as literature reviews, cross-sectional studies, animal model studies and studies that assessed supplementation in pregnant women with thyroid disease were discarded (Supplemental Table 1).

The PICO was defined, namely: Population – pregnant women; Intervention – iodine supplements (iodine supplement use, iodine supplement coverage, iodine content in supplements); Comparator – other children of mothers without iodine supplement use; and Outcomes – development index (mental, psychomotor and verbal), sound response time, IQ (Intelligence Quotient) score (verbal, performance, and reasoning), skills score (language, reading, and writing), mapping test, reading, mathematics and special education.

The scale used to assess neurodevelopment in children selected from the included articles was the Bayley and Children’s Communication Checklist-Short (CCC-S).

The Bayley scale has three indices: mental, psychomotor, and behavioural development. The mental development index assesses the visual perceptual acuity, discrimination between objects, problem solving skills, language, and memory (16-18). The psychomotor development index (PDI) is assessed through postural control and appendicular motricity (16-18). The behavioural development index (BDI) assesses the follow-up of instructions, attitudes, and energy during the test, among other social behaviors (16-18). The Bayley score includes cognition and psychomotor skills with mental index (MDI), with a mean score of 100 (SD 15, range 55-155). The mean language (BDI) score was 100 (SD 15; range 45-155). Severe to moderate neurodevelopmental issues were defined as a mean MDI < 85 or BDI < 85, or both < 70; mild to moderate issues were defined as 85–100, and adequate function was defined as ≥ 100 (19).

However, the CCC-S is effective as a standardized assessment at identifying children with clinically-significant language impairment (20), containing 13 items that best discriminate typically-developing children from peers with language impairment in the validation study (21), with a high degree of internal consistency. Each item provides an example of language behaviour in everyday contexts and covers speech, vocabulary, grammar, and discourse. The items are scored as 0 – absent response, or 1 – present response, with final analysis using statistical methods.

The quality of the studies was assessed according to the checklist of Joanna Briggs Institute (JBI) Critical Appraisal Tools of the Faculty of Health and Medical Sciences at the University of Adelaide, South Australia (22,23). The checklist consider each question should be answered through four options: Yes (Y), No (N), Unclear (U) and Not Applicable (NA). The bias risk percentage calculation is done by the amount of “Y” that has been selected in the checklist. When “NA” was selected, this question was not considered in the calculation, according to the guidelines of JBI. This tool classifies the studies in: up to 49% is considered a high risk of bias. From 50% to 70% is moderate and above 70% is low risk of bias.

## RESULTS

**Figure 1:**
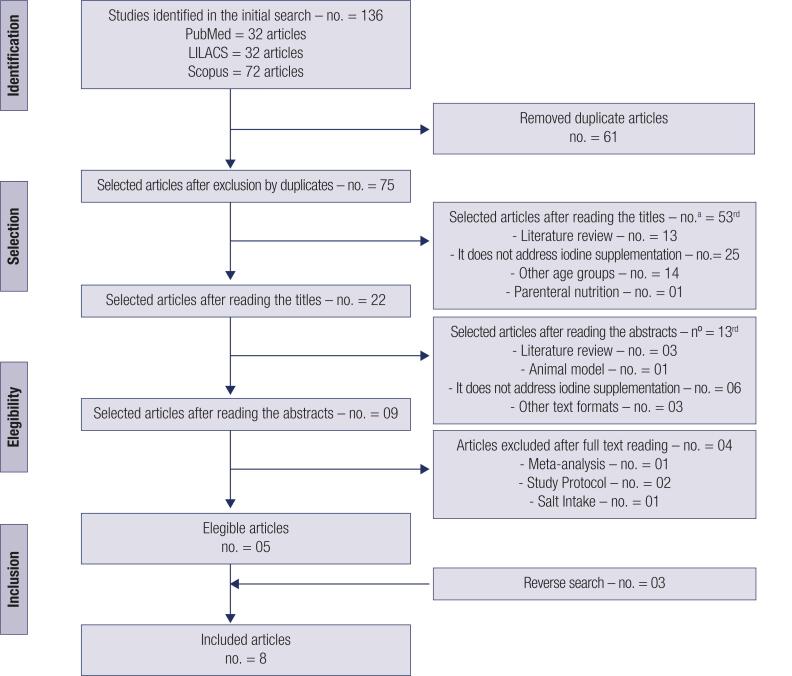
Identification and selection of articles. a. Supplemental Table 1.

The search resulted in 136 articles, of which eight were included in the review (Figure 1 and Supplement Appendix 1). The studies dated from the year 2009 (24) to 2019 (28), four of which were performed in Spain (24,26,27,29), two in Norway (25,28), one in India or Thailand (30), and the other in Australia or New Zealand (31). Two studies were performed in mild to moderate iodine deficiency areas (24,31), five in mild iodine deficiency areas (25-29), and one in a severe iodine deficiency area (29).

Regarding the design, four studies were randomized clinical trials (RTC) (24,26,30,31) and four were cohorts (25,27,28,29). Seven of eight authors used the Bayley scales to assess development of children under 36 months old (24-27,29-31), Gowachirapan and cols. also assessed the IQ of children above 60 months old (30), whereas Abel and cols. used the Children’s Communication Checklist-Short for children between 36 and 96 months old (28) (Table 1).

**Table 1:** Supplementation results in neurocognitive development of children in iodine deficiency areas

Author/Year	Methodology			Main results
	Children by type of mother’s supplementation	Study design	Skills assessed	
Murcia *et al*., 2011 (27)	Spain <100 μg/day of KI (n = 169) 100-149 μg/day (n = 298) ≥150 μg/day (n = 222) Maternal MUIC: NA	Study: Cohort CA: 11-16 months GA: < 12th weeks NA	**Bayley Scales 1^st^ ed.** (16)	**Mild iodine deficiency areas**
			Mental development	↑ in the KI group (≥150 μg), compared to the KI group (<100 and between 100-149 μg)
			Psychomotor development	↑ in the KI group (≥150 μg), compared to the KI group (<100 and between 100-149 μg) ↓ 5,2 scores and ↑ 1,8 odds of a PDI < 85 in the KI group (≥150 μg/day).*
Rebagliato *et al*., 2013 (29)	Spain <100 μg/day of KI 100–149 μg/day ≥150 μg/day Maternal UIC in both group: 102 (71-169) µg/L	Study: Cohort CA: 12-30 months GA: NA	**Bayley Scales 1^st^ ed.** (16)	**Mild iodine deficiency areas**
			Mental development	↑ odds in the KI group (≥150 μg), compared to KI groups (<100 and 100-149 μg) ↓ score in KI group (≥150 μg).
			Psychomotor development	↑ odds in the KI group (≥150 μg), compared to KI groups (<100 and 100-149μg) ↓ score in KI group (≥150 μg).
Markhus *et al*., 2018 (25)	Norway 175 µg/day of KI (n = 155) Placebo (n = 658) 851 pregnant women Maternal UIC: 92 (56-200) µg/L in supplemented group, 77 (50-120) µg/L in control.	Study: Cohort CA: 6 and 18 months GA: 16-26th week	**Bayley Scales 3^rd^ ed.** (18)	**Mild iodine deficiency areas**
			Mental development	↑ in the treated group compared to the placebo group
			Psychomotor Development	↑ in the treated group compared to the placebo group
			Behavior	↓ in the treated group compared to the placebo group
			Verbal IQ (WPPSI – III)	↑ in the treated group compared to the placebo group
Abel *et al*., 2019 (28)	Oslo, Norway 175 µg/day of KI (n = 14,665) Placebo (n = 24,806) 39,471 pregnant women Maternal UIC: 83 (43-138) µg/L L in supplemented group, 59 (32-101) µg/L in control	**Study: Cohort** CA: 36 and 96 months GA: 1-22th week	**CCC-S and CCC-2** (20,21)	**Mild iodine deficiency areas**
			Language skills^a^	↑ in the treated group compared to the placebo group
			Reading skills^a^	↓ in the treated group compared to the placebo group
			Writing skills^a^	↓ in the treated group compared to the placebo group*
			Mapping test Reading^b^	↓ in the treated group compared to the placebo group*
			Mapping test mathematics^b^	↓ in the treated group compared to the placebo group*
			Special education^b^	↓ in the treated group compared to the placebo group*
Velasco *et al*., 2009 (24)	Spain 300 µg/day of KI (n = 133) Placebo (n = 61) Maternal MUIC: 263.0 ± 120.8 µg/L in supplementation, in control: 87.6 ± 62.1 µg/L	Study: Non-randomized controlled trial CA: 3-18 months GA: 8^th^ to 12th week until lactation	**Bayley Scales 2^nd^ ed.** (17)	**Mild to Moderate iodine deficiency areas**
			Mental development	↑ in the treated group, compared to the control group.
			Psychomotor Development	↑ in the treated group, compared to the control group.*
			Behavior	↑ in the treated group, compared to the control group.*
Santiago *et al*., 2013 (26)	Spain Iodized salt (n = 38) 200 µg of KI (n = 55) 300 µg (n = 38) Maternal MUIC: NA	Study: Randomized controlled trial	**Bayley Scales 3^rd^ ed.** (18)	**Mild iodine deficiency areas**
			Mental development	↑ in the control group, compared to the KI group (200 μg), compared to 300
		CA: 6-18 months GA: 10th week	Psychomotor Development	↑ in the control group, compared to the KI group (200 μg), compared to 300
Zhou *et al*, 2015 (31)	New Zealand and Australia 150 μg/day KI (n = 27) Placebo (n = 26) Maternal MUIC: 200 µg/L in supplementation and 150 µg/L in control	Study: Randomized controlled trial CA: 18 months GA: 20th week	**Bayley Scales 3^rd^ ed.** (18)	**Mild to Moderate iodine deficiency areas**
			Mental development	↑ in the placebo group, compared to the treated group.
			Psychomotor Development	↑ in the placebo group, compared to the treated group.
			Behavior	↑ in the placebo group, compared to the treated group.
Gowachirapant *et al*., 2017 (30)	Thailand and India 200 µg/day of KI (n = 303) Placebo (n = 312) 832 pregnant women (T0) Maternal MUIC: NA	Study: Randomized controlled trial CA: 12 and 24 months GA: 14th week CA: 60 and 72 months GA: 14th week	**Bayley Scales 3^rd^ ed.** (18)	**Mild iodine deficiency areas**
			Mental development	↑ in the placebo group, compared to the treated group*
			Psychomotor Development	↔ between groups
			Behavior	↔ between groups
			Sound response time (T).	↑ in the treated group compared to the placebo group*
			Verbal IQ (WPPSI – III)	↑ in the treated group compared to the placebo group
			IQ performance (WPPSI – III)	↑ in the treated group compared to the placebo group
			IQ reasoning (WPPSI – III)	↑ in the treated group compared to the placebo group

MUIC: median urinary iodine concentration; NA: not available; GA: gestational age at the beginning of supplementation; Ed.: edition; KI: potassium iodide; n: sample number; T0: initial time; PDI: Psychomotor Development Index; T.: test; WPPSI-III: 3rd ed Primary Intelligence Scale; IQ: intelligence quotient; CA: Child’s age in the test application; NA: not applicable;

a standardized beta;

b odds ratio.

* Results with statistical significance.

↑ increased;

↓ reduced;

↔ no difference.

The maximum supplementation was 300 μg of potassium iodide (KI) per day (24,26) and one study did not specify supplementation dosages (28). Among the reviewed studies, five started supplementation in the first trimester (24,26,27,29), one in the 14th week (30), another between the 16th and 26th week (25), and one used four different start time categories (28). Only one study continued the supplementation in the lactation period (24); the others finished at the child’s birth (Table 1). Most studies used KI (24,26,30,31); however, some studies did not specify the source of the supplementation (Table 1 and Supplemental Table 2).

The results found an association between supplementation with 150 µg of KI/day and poorer gross motor skills of the PDI standardized beta 0.18 (95% CI: -0.33, - 0.03, p = 0.02) in one study (25), but in another four studies (24,26,27,29) supplementation with ≥ 150 µg of KI/day was associated with a 5.2-point decrease in PDI (95% confidence interval: -8.1, -2.2), decrease in PDI with < 85, odds ratio: 1.7 (95% confidence interval: 1.1, 2.6). The supplementation with 200 or 300 µg of KI/day was related to lower PDI than the iodized salt group. However, another outcome of our study showed that intake of 300 µg of KI/day in breastfeeding was associated with a mean 6.1 ± 0.9 -point increase in PDI compared to the control. Three other studies (28,30,31) did not find an association between iodine supplementation and neurodevelopment in children (Table 1 and Supplemental Table 3).

**Figure 2:**
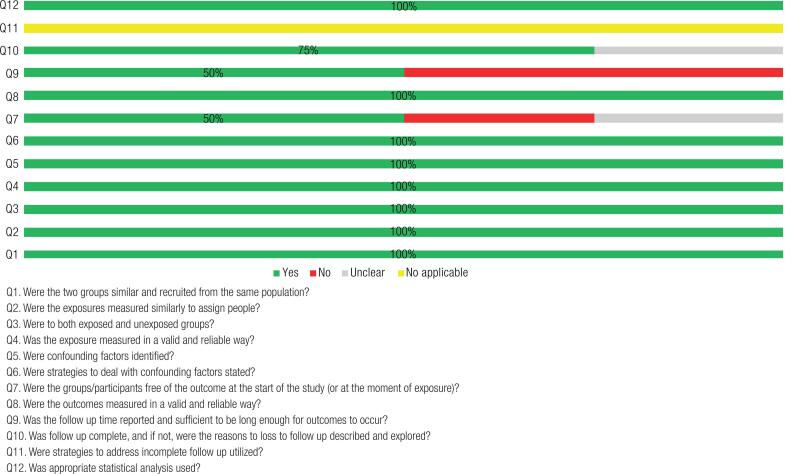
Methodological assessment quality of included studies using Joanna Briggs Institute’s standardized critical appraisal instrument for cohort studies.

**Figure 3:**
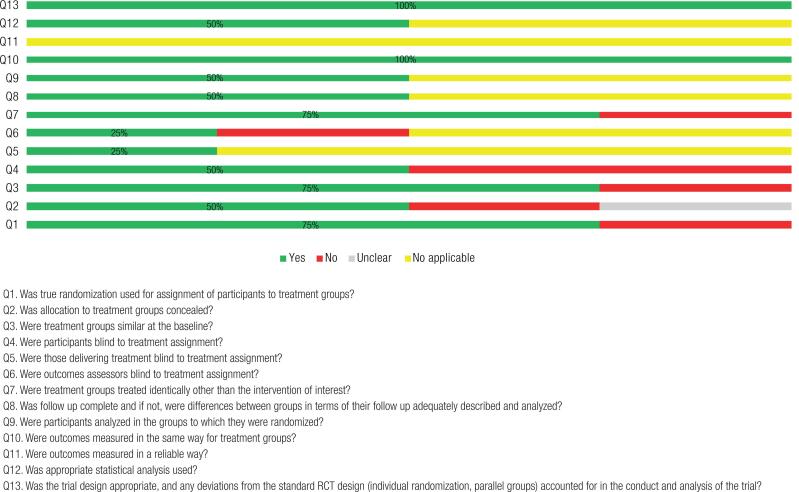
Methodological assessment quality of included studies using Joanna Briggs Institute’s standardized critical appraisal instrument for RCT studies.

Regarding the quality analysis of the studies, the authors observed some limitations in reporting the methods of all trials, leaving some uncertainty in the assessment of several bias criteria, because in two point assessed in RCT studies were high risk of bias (<50%) but, as the studies in many points were moderate or above low risk bias and evidenced a clear delineation of the intervention, as well as were published in good journals we assumed to use all studies include in our review (Figures 2 and 3).

## DISCUSSION

The findings showed an association between iodine supplementation and poor psychomotor development of children aged between 3 and 18 months, living in mild to moderate iodine deficiency areas.

Although not significant, other studies have shown positive results, in which children of supplemented mothers presented higher values of the psychomotor development index (25,27,29,31), behavioural (25,27,29), mental (24,25,30) and communication skills (28), when compared to those who were not supplemented. On the other hand, supplementation of mothers with between 150 and 200 μg of KI per day had no positive effect on the neurocognitive development of their children, as much as in those living in mild as well as moderate iodine deficiency areas, and in some studies the scores were low PDI point and your chance assessed were worse in the treated group (Supplemental Table 2).

None of the RCTs show an association between supplementation and child neurodevelopment, except for a negative association between iodine supplementation and expressive language (BSID) at 1 year in a single trial. The non-RCT studies show mixed results: with a positive association in one case and a negative association in the second. Children in the treatment group were associated with a lower PDI score than in the control group, with a better speaker skills score, poorer skills in the languages domain, lower mapping test results in reading in school, and suboptimal or low scores in mathematics.

Recent evidence has demonstrated these outcomes presented above, showing that 18-month-old children of mothers supplemented with 220-390 μg of KI per day had lower cognitive, language and motor scores (32).

In addition, Gowachirapan and cols. (2017) identified all development scale in primary results with placebo group had higher scores than the treatment group (30) in children aged 12 to 24 months in mild iodine deficiency areas.

Our findings mostly covered children under 24 months old, and the poor psychomotor effect on the children of supplemented mothers was demonstrated in this age group. In our results, the mothers supplemented from the 14th gestational week had a negative association between supplementation and child neurodevelopment, at ages from 14th months.

However, the start of supplementation at the 14th gestational week how showed our findings seem to be late to start supplementation, since the development of the nervous system occurs mainly between the 5-6th gestational weeks and birth, and between birth and 2 years, for infants and children (33,34).

Most of the mothers were supplemented from the 1st trimester of gestation, and in one study, the treatment continued during lactation. Through the results of this study, it was possible to verify that the psychomotor and behavioral development differed significantly among children of mothers supplemented with 300 μg of KI per day, living in areas with mild to moderate iodine deficiency (24). Recommendations from the American Thyroid Association and European Thyroid Association indicate that supplementation started in the pre-gestational period is more effective (6,7).

Supplementation with ≥ 150 μg of KI per day in pregnancy can be improve poor psychomotor development in children. This outcome is observable in lactation if supplementation dose is doubled (300 μg of KI per day).

In another study, children of mothers living in mild iodine deficiency areas and supplemented with 200 μg of KI per day during gestation showed a better response time to sound at 60 to 72 months than their is not supplemented group, but there was no difference between the groups (Supplemental Table 2) (30). This was the only study that used other methods to assess child development beyond the Bayley scale (30), and was the only one that assessed children over two years old, showing that this may be a more interesting time to assess the children’s development. However, use of the Children’s Communication Checklist-Short showed to be better for the assessment of skills and knowledge, including the domains of writing, speaking, reading, mathematical calculations and all languages in older children (>3 years old). This method uses the mental and behavioural skills applicable to the Bayley scale (mental and behaviour development index), and we did not find an association between iodine supplementation and this score in our results (28). These findings were reported for other authors that used the CCC-S to assess older children and used the Bayley score to assess the infant group; they did not find clear differences between these groups (35).

Although the findings showed poor psychomotor development in the children of the supplemented mothers, it seems that this effect is more pronounced in younger children compared to older children using the Bayley scale. However, we observed a high score of the sound response time in children from 60 months, open in this age the children are keen senses.

The use of developmental scales requires caution, since they depend on the evaluator’s observation (36). Despite this, the use of these scales seems to have good results for those living in areas with mild to moderate iodine deficiency. However other factors that may interfere in test results are family income, mother’s education, inadequate urinary iodine concentration (UIC) of the mother, and the presence of siblings, since they directly influence the family stimulus that the child receives (7,11,30,36).

The lack of similarity between initial time, duration, dosage of supplementation, and the time of application of neurocognitive development tests were limiting factors. In addition, three of the seven studies did not assess behavioural development.

The authors observed that supplementation during lactation brings interesting results, which may be the starting point for future research. In areas with mild to moderate iodine deficiency, changes are more likely to develop in children’s psychomotor, behavioural, and mental capabilities. The authors questioned whether the duration of supplementation may have a greater influence than the dose administered, since we did not find any studies with a longer time of supplementation with a lower dose of iodine content, nor did we obtain further assessments of lactation.

The best neurodevelopmental can be good in children with mother living in iodine adequate areas. However, in these results, the mothers in the control group had below adequate UIC, showing iodine deficiency for maternal group in region, which can affect the outcomes in their offspring. Additionally, according to Mao and cols. (2018), the supplementation of pregnant women living in areas of mild iodine deficiency did not have any effect on their children’s neurocognitive development (35).

Improving some factors, such as the start and end times of supplementation, iodine sufficiency of the mothers and the iodine deficiency in the areas where the mothers live, as well as the age of the children and the type of scale used in the tests, can contribute to better results. Therefore, iodine supplementation, if well implemented, can reduce risks to the population and, consequently, reduce public health expenditure.

## Supplement Appendix 1 The full list of search terms used in the literature search

In March 1^st^ to April first week of 2020 The PubMed, LILACS and Scopus databases were searched for relevant articles.

For PubMed (01 RCT and 02 cohort article), LILACS (01 cohort articles) and Scopus database (03 RCT and 01 cohort articles) the following search terms were used; “iodine AND supplementation AND child And development AND cognition ”.

******** *******

For PubMed (03 articles), the following search terms were used: ((iodine) AND (supplementation) AND (child) AND (development) AND (cognition)).

((((“iodine”[MeSH Terms] OR “iodine”[All Fields] OR “iodides”[MeSH Terms] OR “iodides”[All Fields]) AND Supplementation[All Fields]) AND (“child”[MeSH Terms] OR “child”[All Fields])) AND (“growth and development”[Subheading] OR (“growth”[All Fields] AND “development”[All Fields]) OR “growth and development”[All Fields] OR “development”[All Fields])) AND (“Cogn Int Conf Adv Cogn Technol Appl”[Journal] OR “cognitive”[All Fields])

******** *******

For LILACS (01 article), the following search terms were used: “iodine AND supplementation AND child And development AND cognition”.

tw:((tw:(iodine)) AND (tw:(supplementation)) AND (tw:(child )) AND (tw:(development )) AND (tw:(cognitive))))

******** *******

For Scopus database (04 articles), the following search terms were used: “iodine AND supplementation AND child And development AND cognition”

(TITLE-ABS-KEY (iodine) AND TITLE-ABS-KEY (supplementation) AND TITLE-ABS-KEY (child) AND TITLE-ABS-KEY (development) AND TITLE-ABS-KEY (cognition))

Total: 08 articles included

LILACS: 01.

Scopus: 03.

PubMed: 04.

**Supplemental Table 1:** Excluded studies and reasons for exclusion

Reference	Reason for exclusion
A review of the iodine status of UK pregnant women and its implications for the offspring	Is revision
Assessing infant cognitive development after prenatal iodine supplementation	Is revision
Benefit-Cost Analysis in Disease Control Priorities, Third Edition.	Not iodine
Can multi-micronutrient food fortification improve the micronutrient status, growth, health, and cognition of schoolchildren? A systematic review	Is revision
Consequences of iodine deficiency and excess in pregnant women: an overview of current knowns and unknowns	Not iodine
Dietary micronutrients are associated with higher cognitive function gains among primary school children in rural Kenya	Not iodine
Does prenatal micronutrient supplementation improve children’s mental development? A systematic review	Is revision
Driving Policy Change to Improve Micronutrient Status in Women of Reproductive Age and Children in Southeast Asia: The SMILING Project.	Is revision
Effect of iron-, iodine-, and **β**-carotene-fortified biscuits on the micronutrient status of primary school children: A randomized controlled trial	Not pregnant
Effect of micronutrient supplement on health and nutritional status of schoolchildren: Study design	Not pregnant
Effects of iodine supplementation during pregnancy on child growth and development at school age	Supplementation in child
Effects of maternal iodine nutrition and thyroid status on cognitive development in offspring: A pilot study	Not supplementation
Effects of nutrients (in food) on the structure and function of the nervous system: Update on dietary requirements for brain. Part 1: Micronutrients	Not iodine
Effects of nutritional interventions during pregnancy on infant and child cognitive outcomes: A systematic review and meta-analysis	Is revision
Feeding the brain – The effects of micronutrient interventions on cognitive performance among school-aged children: A systematic review of randomized controlled trials	Is revision
Food ingredients and cognitive performance	Not iodine
Growth, development and differentiation: A functional food science approach	Not iodine
Hypothyroxinemia and pregnancy	Not child neurodevelopment
Impact of iodine supplementation in mild-to-moderate iodine deficiency: Systematic review and meta-analysis	Is revision
Iodine as essential nutrient during the first 1000 days of life	Not pregnant
Iodine deficiency and iodine prophylaxis in pregnancy	Is revision
Iodine deficiency in pregnancy and the effects of maternal iodine supplementation on the offspring: a review.,	Is revision
Iodine deficiency in pregnancy, infancy and childhood and its consequences for brain development,	Is revision
Iodine fortification of foods and condiments, other than salt, for preventing iodine deficiency disorders.	Not supplementation
Iodine intake from supplements and diet during pregnancy and child cognitive and motor development: The INMA Mother and Child Cohort Study.	Not intervention
Iodine Nutrition During Pregnancy: Past, Present, and Future.	Is revision
Iodine nutrition in pregnancy and lactation.	Is revision
Iodine plus n-3 fatty acid supplementation augments rescue of postnatal neuronal abnormalities in iodine-deficient rat cerebellum,	Not human
Iodine supplementation improves cognition in iodine-deficient schoolchildren in Albania: A randomized, controlled, double-blind study	Supplementation in child
Iodine supplementation improves cognition in mildly iodine-deficient children	Supplementation in child
Iodine supplementation in pregnancy and its effect on child cognition	Not child neurodevelopment
Iodine: it’s important in patients that require parenteral nutrition.	Not supplementation
Iron deficiency and cognitive functions	Not iodine
Malnutrition, brain development, learning, and behavior,	Not iodine
Maternal iodine status is associated with offspring language skills in infancy and toddlerhood.	Not iodine
Micronutrient adequacy and morbidity: Paucity of information in children with cerebral palsy.	Not iodine
Micronutrient interventions on cognitive performance of children aged 5-15 years in developing countries.	Is revision
Micronutrient supply and health outcomes in children.	Not iodine
Micronutrients in pregnancy in low- and middle-income countries.	Not iodine
Mild iodine deficiency in pregnancy in Europe and its consequences for cognitive and psychomotor development of children: A review,	Is revision
Multiple micronutrient supplementation for improving cognitive performance in children: Systematic review of randomized controlled trials,	Is revision
Neurocognitive outcomes of children secondary to mild iodine deficiency in pregnant women,	Not child neurodevelopment
Nutrient supplementation and neurodevelopment timing is the key,	Not iodine
Nutrition and brain development in early life,	Not iodine
Nutrition and development: Other micronutrients’ effect on growth and cognition,	Not iodine
Nutrition and neurodevelopment in children: Focus on NUTRIMENTHE project,	Not iodine
Nutritional deficiencies and later behavioral development,	Not supplementation
Overall child development: Beyond pharmacological iodine supplementation	Is revision
Prevention and control of iron deficiency anemia amongst young children,	Not iodine
Promoting early child development with interventions in health and nutrition: A systematic review	Is revision
Role of iodine-containing multivitamins during pregnancy for children and brain function: protocol of an ongoing Randomized controlled trial: the SWIDDICH study.,	Not child neurodevelopment
Suggested use of sensitive measures of memory to detect functional effects of maternal iodine supplementation on hippocampal development,	Not in human
Summary of the public affairs committee symposium at the teratology society 2011 annual meeting: The thyroid and iodine: Impacts on pregnancy and child health	Not article
Systemic endocrinopathies (thyroid conditions and diabetes): impact on postnatal life of the offspring.	Not supplementation
Teratology public affairs committee position paper: Iodine deficiency in pregnancy	Not supplementation
The adverse effects of mild-to-moderate iodine deficiency during pregnancy and childhood: A review,	Is revision
The Assessment of Cognitive Performance in Children: Considerations for Detecting Nutritional Influences,	Not iodine
The Effect of Intermittent Antenatal Iron Supplementation on Maternal and Infant Outcomes in Rural Viet Nam: A Cluster Randomized Trial	Not iodine
The effect of iodine supplementation in pregnancy on early childhood neurodevelopment and clinical outcomes: Results of an aborted randomized placebo-controlled trial,	Not child neurodevelopment
The effects of iodine deficiency in pregnancy and infancy,	Not supplementation
The importance of adequate iodine during pregnancy and infancy,	Not iodine
The influence of dietary status on the cognitive performance of children,	Not iodine
The role of nutrition in children’s neurocognitive development, from pregnancy through childhood,	Is revision
Therapy of endocrine disease: Impact of iodine supplementation in mild-to-moderate iodine deficiency: systematic review and meta-analysis.	Is revision
Thyroglobulin level at week 16 of pregnancy is superior to urinary iodine concentration in revealing preconceptual and first trimester iodine supply.	Is revision
Thyroid and iodine nutritional status: a UK perspective.	Is revision
Thyroid-stimulating hormone (TSH) concentration at birth in Belgian neonates and cognitive development at preschool age.	Not supplementation
What do we know about iodine supplementation in pregnancy?	Is revision
Biomarkers of Nutrition for Development (BOND): Vitamin B-12 Review”,2018	Review and not iodine
Iodine as essential nutrient during the first 1000 days of life”,2018	Not pregnant
Iodine intake from supplements and diet during pregnancy and child cognitive and motor development: The INMA Mother and Child Cohort Study”,2018	Not iodine intake
Iodine supplementation for the prevention of mortality and adverse neurodevelopmental outcomes in preterm infants. 2019	Review
Mild-to-moderate gestational iodine deficiency processing disorder”,2019	Review
Role of iodine-containing multivitamins during pregnancy for children’s brain function: Protocol of an ongoing randomized controlled trial: The SWIDDICH study”,2018	Protocol
Supplementing mothers and their offspring with long-chain ω-3 PUFAs offers no benefit compared with placebo in infant development”,2019	Not iodine
The effect of iodine deficiency during pregnancy on child development”,2019	Review
Thyroid function in preterm infants and neurodevelopment at 2 years. 2020	Direct supplementation in infant
WITHDRAWN: Iodine supplementation for preventing iodine deficiency disorders in children. Nov. 2018	Review
	**62**

**Supplemental Table 2:** Description of the interventions applied in the evaluated studies

Author/ Year	Dosage	Supplementation interval	Form of supplementation
Velasco *et al*., 2009^24^	300 µg/day of iodine	≤13^th^ week of pregnancy to lactation	Potassium iodide (KI)
Murcia *et al*., 2011^27^	<100 µg/day	≤13^th^ week of pregnancy to delivery	Different supplementary sources of iodine
	100–149 µg/day		
	≥150 µg/day		
Santiago *et al*., 2013^26^	200 µg/day	≤10^th^ week of pregnancy to delivery	KI
	300 µg/day		
Rebagliato *et al*., 2013^29^	<100 μg/day of KI	≤13^th^ week of pregnancy to delivery	KI or vitamin/mineral preparations containing iodine
	100– 149 μg/day		
	≥150 μg/day		
Zhou *et al*., 2015^31^	150 μg/day	≤20^th^ week of pregnancy to delivery	KI
Gowachirapant *et al*., 2017^30^	200 µg/day	≤14^th^ week of pregnancy to delivery	KI
Markhus *et al*., 2018^25^	150 – 200 µg/day	≤26^th^ week of pregnancy to delivery	Different supplementary sources of iodine
Abel *et al*., 2019^28^	NA	Week 0-26 before pregnancy	Different supplementary sources of iodine
		GW: 0–12	
		GW: 12–22	

KI- Potassium iodide; GW: Gestational Week; NA- Not available.

**Supplemental Table 3:** Effects of iodine supplementation in pregnancy on neurocognitive development on offspring

Trial	Iodine Status of region	Mental development (MDI)	Psychomotor development (PDI)	Behaviour development (BDI)	Sound response time (T).	Verbal IQ (WPPSI – III)	IQ performance (WPPSI – III)	IQ reasoning (WPPSI – III)
Velasco *et al.*, 2009(24)	Maternal MUIC during pregnancy (last third trimester): MUIC 263.0 ±120.8 µg/L iodine (n=133) > control 87.6 ± 62.1 µg/L (n=31, Iodine in breast milk: within 3.6 ± 2.9 months of lactation, intake 1.8 ± 1.2 µg/100 mL, in 300 µg/day of supplemented group (n = 67), vs. 1.4 ± 0.6 µg/100 mL, without supplement in control group (n=21). Infant MUIC: 203.5 ± 150.6 µg/L iodine, in 300 µg/day supplemented group (n= 75) > control 114.6 ± 60.9 µg/L iodine (n= 30) Southern of Spain (Malaga and Osuna in Andaluzia state) is areas of mild to moderate iodine deficiency and iodine deficiency in pregnant	Infant: NA	Maternal intake of 300 µg/day, that were breastfeeding compared with without group (n= 19) associated with a 6.1+0,9 point high in PDI with control. High PDI in child was associated also with lower FT4 in third trimester of pregnancy in mother in supplemented group (r: 0.50; P <0.0001).	Mother intake 300 µg/day of iodine in supplements as compared with the control group was seems more in agreement with for the following items: To reaction to the mother, Odds reaction: 4.3 (CI 95%; 1.69 –10.86, P <0.05), Cooperation, Odds reaction: 17.29 (CI 95%; 2.00 –148.86) 0.009 22.45, P<0.008), activity, Odds reaction: 8.55 (CI 95%; 1.64–44.44, P<0.01), and producing sounds by banging, Odds reaction: 9.00 (CI 95%; 2.66 –30.44) (P <0.001).	NA	NA	NA	NA
Murcia *et al.*, 2011 (27)	Maternal MUIC during pregnancy: NA. Infant MUIC.: NA Valencia, is north of Spain,there does not know of iodine situation. But recently evidence (INNA Project) showed an increased risk of raised thyroid stimulating hormone (TSH) during the first half of pregnancy for women who consumed iodine supplements, because in some years ago there were iodine deficiency	Infant: NA	Maternal intake of ≥150 mcg/day, compared with <100 mcg/day, of iodine from supplements was associated with a 5.2- point decrease in PDI (95% confidence interval: -8.1, -2.2) and a 1.8-fold increase in the odds of a PDI <85 (95% confidence interval: 1.0, 3.3)	NA	NA	NA	NA	NA
Santiago *et al.*, 2013 (26)	Maternal MUIC during pregnancy: NA MUIC least 1 year before becoming pregnancy: 177.1 + 82.3 µg/L in 200 µg group (n=55) and 222.0 +85.5 µg/L in 300 µg/L in supplemented group (n=38), with control 130.2 + 64.8 µg/L (n=38) Iodine in breast milk: Not diff. in groups but all is >100 µg/L: in treatment groups is high intake: 2.4·+1.2 µg/100ml in 200 µg group, 2.2+1.5 µg/100ml in 300 µg group, that in control group: 1.4+0.9µg/100ml Infant MUIC: NA Objective of this study was assessment iodine intake and their consequences in health in area with iodine deficiency	Maternal supplementation with 200 or 300 µg/day was related with lower PDI than the iodized salt group (NA). The results showed that the age at which this psychometric test was performed correlated significantly with a scales MDS (r 0·93,P,0·001) and the PDS (r 0·90, P,0·0001), but not with indices (MDI and DPI). The showing a age to be important factor of measured its testes.		NA	NA	NA	NA	NA
Rebagliato *et al*., 2013 (29)	Maternal MUIC during pregnancy: Median in three municipality of Valencia, in both groups is Asturias 102 (71–169) µg/L (n = 412) Gipuzkoa 169 (108–284) µg/L (n = 548) Sabadell 90 (52–148) µg/L (n = 559) Infant MUIC: NA In Asturias and Gipuzkoa is iodine sufficiency areas and Sabadell is several. But the supplementation iodine program is implemented in both because the author wanted to see effect of neurodevelopment on infant in sufficiency and deficiency iodine consumer.	A negative association of iodine supplementation with low development. No difference with groups, site of study and user of iodized salt or supplement. But consumption during pregnancy of 150 μg/day or more of iodine was related to a decrease in Psychomotor development score, although only significantly so in Asturias less than 85 (OR = 1.7, 95% CI: 1.1, 2.6).		NA	NA	NA	NA	NA
Zhou *et al.*, 2015 (31)	Maternal MUIC during pregnancy 3rd trimester: 200 µg/L in supplemented group > (n=27), with 150 µg/L (n=26) in control Iodine in breast milk: 6 weeks after birth was 1.1 (0.8–1.5) μg/100ml in both groups. 150 µg/day of KI (n=29) for supplemented group, with without iodine supplements in control (n=30) Infant MUIC: NA Australia and New Zeland are moderate iodine deficiency areas.	Infant: NA	Infant: NA	Infant: NA	NA	NA	NA	NA
Gowachirapan *et al*., 2017 (30)	Maternal MUIC during pregnancy: NA 200 µg/day of KI group (n=303) Placebo (n=312) Infant MUIC: NA In INNA Study, the authors found in a sample of women from the Valencia region, an iodine-sufficient areas, that maternal consumption of multivitamins containing iodine was related to lower psychomotor achievement of their infants at 1 year of age.	All the scores in the primary outcomes were higher in mean in the placebo than in the intervention group (not significant).			Infant: no diff iodine vs. control (n=unclear, means not presented)	Infant: A negative association of iodine supplementation with expressive language (BSID) at 1 year in the first one	Infant: NA	Infant: no congenital goitre was found in either group
Markhus, M. W., 2018 (25)	Maternal MUIC during pregnancy 1^st^ trimester: Median 92 (56,200) µg/L in supplemented group (n=658) > and 77 (50,120) µg/L in control (n=155). In total: 79% of women had a MUIC < 150 µg/L and 28% < 50 µg/L. 175 µg/day of KI (n=155) for supplemented group, with without iodine supplements in control (n=658). Infant MUIC: NA	Infant: NA	Supplementation with 150 µg/day was associated with poorer gross motor skills, standardized beta = - 0.18 (95% CI = -0.33, - 0.03, p = 0.02).	NA	NA	Infant: NA But women having a low MUIC in pregnancy (50> but <100 µg/L) was significantly associated with poorer skills in language domains (receptive and expressive) in infancy and toddlerhood.	NA	NA
Abel M.H. al., 2018 (28)	Maternal MUIC during pregnancy 1st and 2nd trimester: Median 83 (43, 138) µg/L in supplemented group (n= 14,665) > and 59 (32, 101) µg/L in control (n= 24,806). 175 µg/day of KI (n=14,665) for supplemented group, with without iodine supplements in control (n=24,806). Infant MUIC: NA	NA The results are consistent in demonstrating no beneficial effects of iodine supplement use in pregnancy. Not have any associations between maternal use of iodine-containing supplements and child outcomes. But in sample, 6.9% of the 8year old were granted special education at school. According to the mother, 28% had suboptimal or low score on the mandatory mapping test in school in reading, and 18% had suboptimal or low score in mathematics.			NA	NA But in control: Materna iodine intake was associated with child language skills at age 3 years. children with mild to moderate language delay at 3 years (3.1%) scored on average 1.2 SD higher on the language score (CCC-S) at 8 years (95% CI 1.1, 1.3), and those with severe language delay at 3 years (0.7%) scored 2.8 SD higher (95% CI 2.3, 3.2). Higher scores at 8 years indicated poorer language skills.	NA	NA

NA, Not available; MUIC, median urinary iodine concentration; n, number; KI, iodide potassium; No diff, no difference; UI, urinary iodine; CCC-S: Children’s Communication Checklist-Short.
